# Laparoscopic management of superior mesentric artery syndrome: A case report and review of literature

**DOI:** 10.4103/0972-9941.43092

**Published:** 2008

**Authors:** Ramesh Makam, Tulip Chamany, Vamsi Krishna Potluri, Prashanth J Varadaraju, Rajesh Murthy

**Affiliations:** Department of Minimal Access Surgery, A.V. Hospital, Bangalore, India

**Keywords:** Abdominal doppler scan, feeding jejunostomy, intestinal obstruction, laparoscopy, laparoscopic duodeno-jejunostomy, Sma syndrome

## Abstract

Superior mesentric artery syndrome is a rare cause of high small bowel obstruction, caused by compression of the transverse part of the duodenum in between the superior mesentric artery and aorta. Patients present with chronic abdominal pain, vomiting and weight loss. We report a case of superior mesenteric artery syndrome, managed laparoscopically with laparoscopic duodenojejunostomy.

## INTRODUCTION

Superior mesenteric artery syndrome is a rare condition that may cause high small bowel obstruction. This is caused by compression of the fourth (transverse) part of the duodenum between the superior mesenteric artery and the aorta. Traditionally, this condition has been treated by an open duodeno-jejunostomy. We report a patient with superior mesenteric artery syndrome managed successfully by laparoscopic duodeno-jejunostomy.

## CASE REPORT

A 14-year-old girl presented with complaints of worsening pain abdomen, abdominal fullness and loss of weight of nine months duration. She had recurrent attacks of colicky pain in epigastrium, associated with nausea and decreased appetite. Her symptoms increased postprandially and were relieved by lying laterally or in knee-elbow position. There was a history of significant weight loss. She lost 11 kg over the last nine months and had a BMI of 11. She was severely malnourished. There was no palpable mass or organomegaly on abdominal examination. Initial laboratory blood tests including complete blood count, liver function tests, urea and electrolytes were normal.

**Figure 1:**
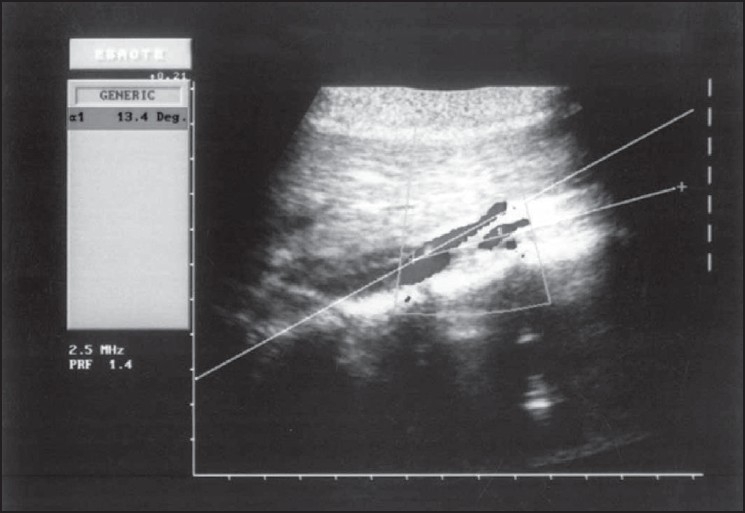
Abdominal Doppler ultrasound demonstrating aortomesentric angle of 13 degrees

**Figure 2:**
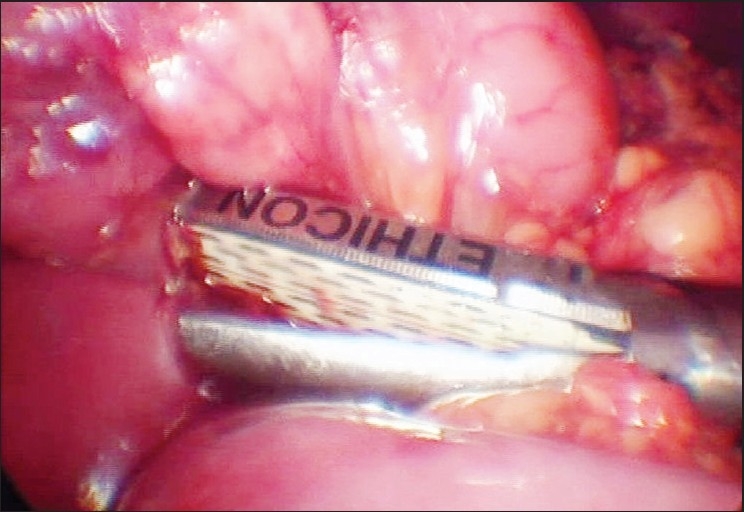
Laparoscopic duodenojejunostomy using a GIA stapler

Abdominal radiograph revealed a dilated stomach with a prominent air fluid level. Endoscopic examination of the upper gastrointestinal tract revealed narrowing of the third part of the duodenum due to extrinsic compression. Barium meal follow-through revealed dilated stomach (extending into the pelvic cavity), dilated second and third parts of the duodenum with to and fro peristalsis. Extrinsic pressure effect was noted at the junction of the third and fourth parts of duodenum. The aortomesentric angle was 13 degrees on abdominal doppler ultrasound [Figure 1]. The clinical presentation in conjunction with these studies established the diagnosis of SMA syndrome. She was given strict advice on adequate nutrition and was recommended proper positioning after eating e.g. left lateral decubitus, prone or knee elbow position, which resulted in decrease in the progressive weight loss, but she continued to have pain and aversion to food. As conservative treatment failed, laparoscopic duodenojejunostomy [Figure 2] was planned. Primary trocar (10 mm) was placed at the umbilicus by Hasson's technique. Four additional trocars were placed. On laparoscopic examination, second and third parts of the duodenum were distended. Jejunum was normal. Examination of the rest of the abdomen was normal. After kocherisation of the duodenum, a proximal loop of jejunum was anastamosed to the third part of duodenum using a 45 mm endo GIA stapler. Feeding jejunostomy was done. The procedure was technically difficult in this patient due to the small size of the abdomen, which was mainly due to emaciation. The operative time was 180 min. She had no postprandial pain postoperatively when she was started on liquid diet on the 3^rd^ postoperative day. She was discharged on the 5^th^ postoperative day. She is on regular follow-up since six months and has gained 10 kg.

## DISCUSSION

SMA syndrome was first described by Professor Carl von Rokitansky in 1842.[1] Wilkie[2] published the first series of 75 patients in 1927, henceforth SMA syndrome is also called Wilkie's syndrome. Cast syndrome, arteriomesentric duodenal compression, aortomesentric artery compression, duodenal vascular compression are the other terms used to describe the same condition.

Normally, SMA forms an angle of approximately 45 degrees (range, 25 - 60 degrees) with the abdominal aorta and the aortomesentric distance is 10 to 28 mm.[3] The retroperitoneal fat and lymphatic tissues serve as a cushion, holding the SMA off the spine and protect the duodenum from vascular compression.[4] In SMA syndrome, the aortomesentric angle is narrowed to 6 to 15 degrees and the aortomesentric distance is reduced to 2 to 8 mm.[5] The narrowed aortomesentric angle causes extrinsic compression on the third part of the duodenum, which crosses in between the SMA anteriorly and aorta posteriorly.

The narrowed aortomesentric angle can be due to anomalous anatomy such as high insertion of duodenum at the ligament of Treitz, low origin of the SMA or due to loss of retroperitoneal fat causing a reduction in the angle. Loss of retroperitoneal fat due to weight loss is the pathogenesis of SMA syndrome in patients with malabsorption, anorexia nervosa, extensive burns, major surgical procedures, cast immobilisation and severe traumatic brain and spine injuries.

Patients with SMA syndrome often present with chronic symptoms of recurrent epigastric pain, bilious vomiting, early satiety and loss of weight. These symptoms are aggravated post-prandially and are relieved by lying in left lateral or knee- elbow position. Acute presentation is seen in patients who have suffered a recent rapid weight loss (spinal cord injuries, extensive burns).

Barium study demonstrates dilated stomach, second and third parts of the duodenum with cut off in the third part and to and fro peristalsis. Narrowing of the third part of the duodenum due to extrinsic compression is seen on upper GI endoscopy. Aortomesentric angle of less than 25 degrees is confirmed by Doppler USG (13 degrees in our case), CT abdomen or MRA.

Patients with acute SMA syndrome often respond to conservative treatment.[6] Conservative initial treatment includes nasogastric decompression, correction of dehydration, prevention of electrolyte imbalance. In severe cases, nasojejunal feeding or total parenteral nutrition is effective to prevent further weight loss. Prokinetic drugs (cisapride, metaclopramide) may be helpful.

Most of the patients with chronic SMA syndrome require surgical intervention.

Surgery is indicated in patients with:

- failed conservative treatment
- longstanding disease with progressive weight loss and duodenal dilatation with stasis
- complicated peptic ulcer disease secondary to biliary stasis and reflux

Duodenojejunostomy, Gastrojejunostomy, Strong's procedure[7] (lysis of ligament of Treitz) are the surgical options in treatment of SMA syndrome. Duodenojejunostomy is the most effective of the procedures, with a success rate of 90%.[8] The invasive nature of this treatment is a drawback, especially in debilitated patients. Laparoscopic Duodenojejunostomy, first described by Gersin and Heniford in 1998,[9] offers a new minimally invasive therapeutic approach to SMA syndrome.[10] We have observed that laparoscopic duodenojejunostomy has the benefits of minimal postoperative pain, short hospital stay and early return to normal activities.
